# “Pharmacotrophy”: a playful tournament for game- and team-based learning in pharmacology education - assessing its impact on students’ performance

**DOI:** 10.1186/s12909-024-05157-z

**Published:** 2024-03-01

**Authors:** Clément Delage, Maeva Palayer, Dominique Lerouet, Valérie C. Besson

**Affiliations:** 1 Université Paris Cité, Inserm, Optimisation Thérapeutique en Neuropsychopharmacologie, F-75006 Paris, France; 2 Service de Pharmacie, AP-HP, Hôpital Lariboisière-Fernand Widal, F-75010 Paris, France; 3 Unité Pédagogique de Pharmacologie, Faculté de Pharmacie de Paris, Université Paris Cité, F-75006 Paris, France

**Keywords:** Pedagogical innovation, Pharmacology education, Education research, Game-based learning, Competition-based learning, Pharmacotrophy

## Abstract

**Background:**

At the Faculty of Pharmacy of Paris, we conducted a pharmacology tournament in 2021 and 2022, named “Pharmacotrophy”, to offer a game-, team- and competitive-based learning innovation based on fun and challenge. This article aims to (1) provide a detailed overview of the organisation of “Pharmacotrophy,” (2) present and compare feedback from both students and teachers, and (3) assess the impact of student participation on their exam marks.

**Methods:**

“Pharmacotrophy” took place in 2021 and 2022 over a two-week period at the beginning of the exam revision phase. It involved a combination of remote matches using the online quiz creation tool Kahoot!® and in-person matches. Teams, consisting of three students from the 4th or 5th year, participated in several selection rounds leading up to the final match. The questions covered various topics from the pharmacology curriculum. Using an anonymous online survey, we collected the feedback from students and teacher regarding the organisation of the tournament and the interest and difficulty of the different type of questions. We retrospectively compared the exam marks of 4th year students who took part in “Pharmacotrophy” (n_2021_ = 19 and n_2022_ = 20) with those of the rest of the 4th year (n_2021_ = 315–320 and n_2022_ = 279–281), both in the year before “Pharmacotrophy” and just after the tournament.

**Results:**

Students highlighted the educational benefits of team-based and game-based learning. This novel approach positively and constructively motivated students to review pharmacology. Additionally, students appreciated the establishment of a trust-based relationship with their teachers. All students had a similar pharmacology level based on their exam results in the year before “Pharmacotrophy.” After the tournament, participants had marks 20.1% higher in pharmacology questions compared to non-participants (*p* = 0.02), while they had comparable overall levels, as evidenced by their final grade averages and marks in non-pharmacology questions. Moreover, participants who advanced further in the competition achieved higher marks in pharmacology questions compared to those who were eliminated early in the tournament.

**Conclusion:**

The implementation of “Pharmacotrophy” provided students with an enjoyable way to review pharmacology coursework and revived the interest in pharmacology for some. Specifically, participating in “Pharmacotrophy” led to an increase in pharmacology marks for students who were not among the top performers in the class or did not excel in pharmacology in the previous year. This study quantified the pedagogical value of this innovative curriculum in terms of knowledge acquisition.

**Supplementary Information:**

The online version contains supplementary material available at 10.1186/s12909-024-05157-z.

## Background

In recent times, conventional teacher-centred courses have displayed limitations, as students have shown a preference for diverse learning methods [1–3]. The advancement of new computer and interactive technologies in education, accelerated by the emergence of Covid-19 [4], has paved the way for varied educational approaches [5]. The use of game elements (e.g., points, leader boards, prizes) in non-gaming contexts is referred to as gamification or “gamified learning” [6]. The term “gamification” was introduced in 2011 [7] and was first conceptualized by Morris and colleagues in 2013 [8]. Since then, the number of studies relating to gamification has experienced an exponential growth [9–11].

Transferring game dynamics to the educational field aims to foster changes in learning behaviours or attitudes towards learning [6], to foster students’ motivation, grades and relationship with the curriculum and teachers [11, 12] and to promote knowledge acquisition in a dynamic way [13, 14]. Although there is no established list or consensus in the theories underlying the benefits of gamification [6], some suggested theories include:

- Experiential Learning: exposing students to a concrete experience encourages them to reflect and to change their behaviour accordingly. Gamification seeks to promote active engagement through interactive experiences that can reflect the students’ own experience [15].
- Self-Determination: learners are more motivated and engaged when they feel autonomous and in control of their learning. Gamification fosters autonomy by providing choices and opportunities for students to define their own learning needs, goals and trajectory [16].
- Reinforcement Learning: learners are more likely to repeat behaviours and actions that are followed by a reward or positive feedback, and less likely to repeat behaviours that are followed by punishment or negative feedback. Gamification can leverage this principle by using rewards and penalties to encourage students to engage in desired behaviours and in acquiring specific knowledge [17].
- Deliberate Practice: expertise is developed through focused and intentional practice. It is characterized by several key features, including the use of challenging tasks, the provision of immediate feedback, and the repetition of skills and behaviours until they become automatic. Gamification allows learners to improve their skills and knowledge through repeated and goal-directed interactions with the game environment [18].
- Social Comparison Learning: learning is influenced by observation, modelling and feedback from others. Gamification can encourage collaboration and competition among students, as well as providing opportunities for students to learn from and give feedback to their peers [19].

From a neurobiological perspective, gamification can increase the dopamine levels in the brain, especially through rewards, pleasure and fun [20–22]. Dopamine plays a major role in motivation, associative learning and working memory, primarily through neurogenesis and an increase in synaptic plasticity [23–25]. Thus, the involvement of the reward system and the mesolimbic pathway promotes learning [22, 26, 27].

Several recent studies have demonstrated the positive impact of gamification on learning, especially in the healthcare field. Gamification represents a major lever to foster students’ motivation [6, 11, 13]. It also promotes players’ cognitive, psychomotor, and emotional competencies [28]. Furthermore, gamification has been shown to be effective in improving learning and knowledge retention in healthcare [6, 29]. Van Gaalen and colleagues also found that the inclusion of competition often led to improved learning outcomes, such as increased knowledge retention, skill development, and positive changes in behaviour [6]. This supports the theory of competitive-based learning (CBL), which involves introducing elements of competition and challenge in learning to promote knowledge acquisition [30].

These concepts have motivated the development of game-based learnings (GBL) and serious games, especially in medical [6, 31, 32] and pharmaceutical [33–36] curricula, such as role-playing games [37], TV-show games [38–40], card games [41–43], crossword puzzles [44], mobile applications [45], augmented reality [46] and tournaments [47–49]. While several studies have shown the positive effect of educational innovations in healthcare professional training, especially those based on competition, most of them have only focused on satisfaction or knowledge acquisition using pre-post studies without control group or randomisation [50–53]. Only a few compared the benefits of the new pedagogical approach on the participating students to a control group or to the previous method [49].

Compared to other medical disciplines, pharmacology poses a challenge due to the extensive amount of material that needs to be memorized. Maintaining learners’ motivation and concentration is particularly difficult for pharmacology teachers, making learner-centred and active learning methods all the more beneficial [54]. Students are keen for this and they find that online quiz solutions such as Kahoot!® help them to retain knowledge [55]. This explains the proliferation of gamification in pharmacology in recent years [28].

With these considerations in mind, the pharmacology chair of the Faculty of Pharmacy of Paris led a game-, team- and competition-based educational initiative called “Pharmacotrophy”. This initiative is a team tournament where pharmacy students engage in a friendly competition by answering pharmacology questions through live online quiz. “Pharmacotrophy” aims to incorporate various educational theories including, self-determination, reinforcement learning, and social comparison learning. The objectives of this tournament were (1) to motivate students to revise pharmacology, and (2) to promote the learning of pharmacological knowledge.

This article provides a detailed overview of the organisation of the tournament. To assess the achievement of the first objective, we report and compare the feedback from students and teachers who organised “Pharmacotrophy” regarding (1) its organisation, and (2) the difficulty and the interest of the different type of question. To evaluate the achievement of the second objective, we compare exam marks before and after “Pharmacotrophy” between participants and other students in the same class.

## Methods

### Overview of the pharmacy program

In France, Doctor in Pharmacy degree (PharmD) is awarded after a minimum of 6 years of training [56]. At the Faculty of Pharmacy of Paris, fundamental pharmacology is taught during the first semester of the 3rd year, while therapeutic pharmacology is taught during the second semester of the 3rd year (cardiovascular diseases, cardiovascular risk factors and digestive system diseases) and during the 4th year (pain and inflammation, psychiatry, neurology, infectious diseases).

Each class at the Faculty of Pharmacy of Paris consists of approximately 300 to 350 students.

### Overall organisation

“Pharmacotrophy” tournament was scheduled during the exam revision period, taking place after the completion of the last 4th year courses and before the exams.

“Pharmacotrophy” was designed specifically for 4th year students for two reasons. First, since they completed the entire pharmacology curriculum by the end of the year, it allowed us to diversify questions by covering a wider range of topics. Secondly, 4th year students must pass an oral exam that covers all the disciplines (including pharmacology) and synthesizes all the pharmaceutical knowledge acquired since their 1st year. This makes them highly motivated to find effective ways to revise the entire curriculum. As the questions could cover field they had not yet studied, the competition was not open to students in 1st, 2nd and 3rd years. While a separate competition for 3rd year students with adapted questions could have been designed, it would have required additional time and resources. Although 5th- and 6th-year students were allowed to participate, they were not the primary target audience. Students formed their own team of 3 individuals and registered as a team. “Pharmacotrophy” was designed, set up, organised and run by 3 pharmacology teachers from the Faculty of Pharmacy of Paris (VCB, DL and CD) in 2021 and an additional teacher (MP) in 2022. The Faculty of Pharmacy of Paris provided support in promoting the event through social networks and in obtaining goodies from the “Université Paris Cité” for participants and winners.

### Tournament design

**Fig. 1:**
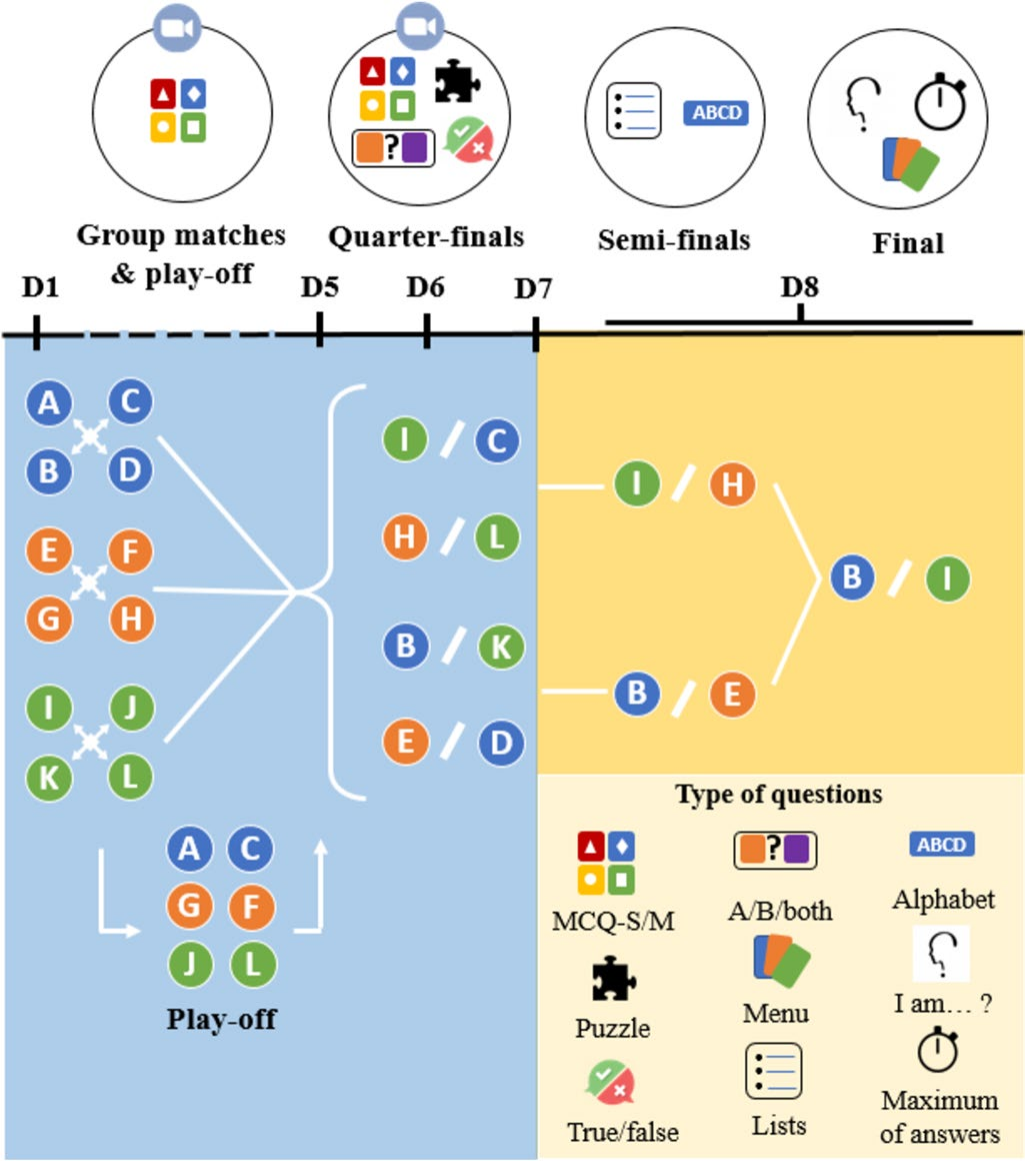
2022 “Pharmacotrophy” overall design

The first week was dedicated to group stage and the second week to the knockout stage. Matches took place from 6:30 pm to 7:30 pm. The overall organisation of the 2022 edition is presented in Fig. 1 and detailed organisation of 2021 and 2022 editions can be found in Additional file 1.

In the 2021 group stage, teams were distributed into three groups of three teams. Each team played twice with opposing teams. The two best teams of each group qualified for the quarter-finals which consisted in three matches in a two-legged format over 2 days. Then, the three qualified teams played each other simultaneously in a single semi-final. At last, the two best teams met the last day for the final.

In the 2022 group stage, teams were distributed into three groups of four. Each team faced other teams once, resulting in three group matches for each team. The two best teams from each group qualified for the quarter-finals. The remaining teams competed in a 6-team play-off match and the two best teams from this play-off also qualified for the quarter-finals. Knockout stage matches consisted in single matches.

Due to the SARS-COV-2 pandemic lockdown in France, the entire 2021 tournament was conducted remotely. In 2022, the group stage matches and the quarter-finals were also held remotely, but the semi-finals and the final were face-to-face matches that took place at the Faculty of Pharmacy of Paris.

### Matches and questions

#### Organisation

Online matches were held online via Zoom® software and all students (participants or not) could attend matches. The Kahoot!® online website was used to present questions, and participants answered using the Kahoot!® mobile app or website on their own smartphones or computers. Players could participate using pseudonyms, and the answers were not displayed to the audience. After each question and at the end of the match, only the scores of the top three players were shown.

Correct answers were provided after each question, but they were not explained for two reasons. First, the aim of “Pharmacotrophy” was not to provide a pharmacology course, and giving explanations could disrupt the fun and dynamic nature of the tournament. Moreover, not providing explanations encouraged participants to seek the information themselves and actively engage in revision. Additionally, some questions could be similar or on identical themes from one match to another. Thus, the explanation of an answer could have given the answer to a subsequent question.

#### Score calculation

For remote matches, each participant individually provided their answers and received points for each correct response. The team score was calculated as the sum of each team member’s individual score at the end of the match. For face-to-face matches, the entire team collectively provided the answers. Points for correct answers were added up within different games to obtain the final score for the team.

#### Question redaction

Questions were individually written by the four pharmacology teachers in charge of “Pharmacotrophy” and organised by topic in an online question bank using Google Drive®. The day before matches, one teacher randomly selected the appropriate number of questions from the question bank while ensuring a balanced representation of topics. Subsequently, all teachers reviewed the questions in a dedicated meeting to ensure their relevance and validate the correct answer. Any irrelevant or overly difficult questions, as well as any incorrect answers, were changed.

#### Question topics

Questions were elaborated on the following topics:

- fundamental pharmacology (ligands and receptors, molecular and cellular responses, neurotransmissions, pharmacometry);
- cardiovascular diseases (hypertension, heart failure, rhythm disorders, angina, infarcts);
- cardiovascular risk factors and digestive system pathologies (diabetes, dyslipidaemia, diarrhoea, constipation, vomiting);
- pain and inflammation (stage I, II and III of pain, acute and chronic inflammatory diseases such as arthritis, osteoarthritis, rheumatoid arthritis and gout);
- psychiatry (depression, anxiety, bipolar disorder, sleep disorders, psychosis);
- neurology (epilepsy, Parkinson’s and Alzheimer’s diseases, multiple sclerosis);
- infections (bacterial, viral and fungal infections).

Within these topics, questions covered information about the diseases (pathophysiology, aetiologies, treatment guidelines, primary and secondary prevention) or the drugs used for treatment (mechanism of action, pharmacokinetic, side effects, precautions for use, drug interactions, indications and usage) (Tables 1 and 2).

**Table 1:**
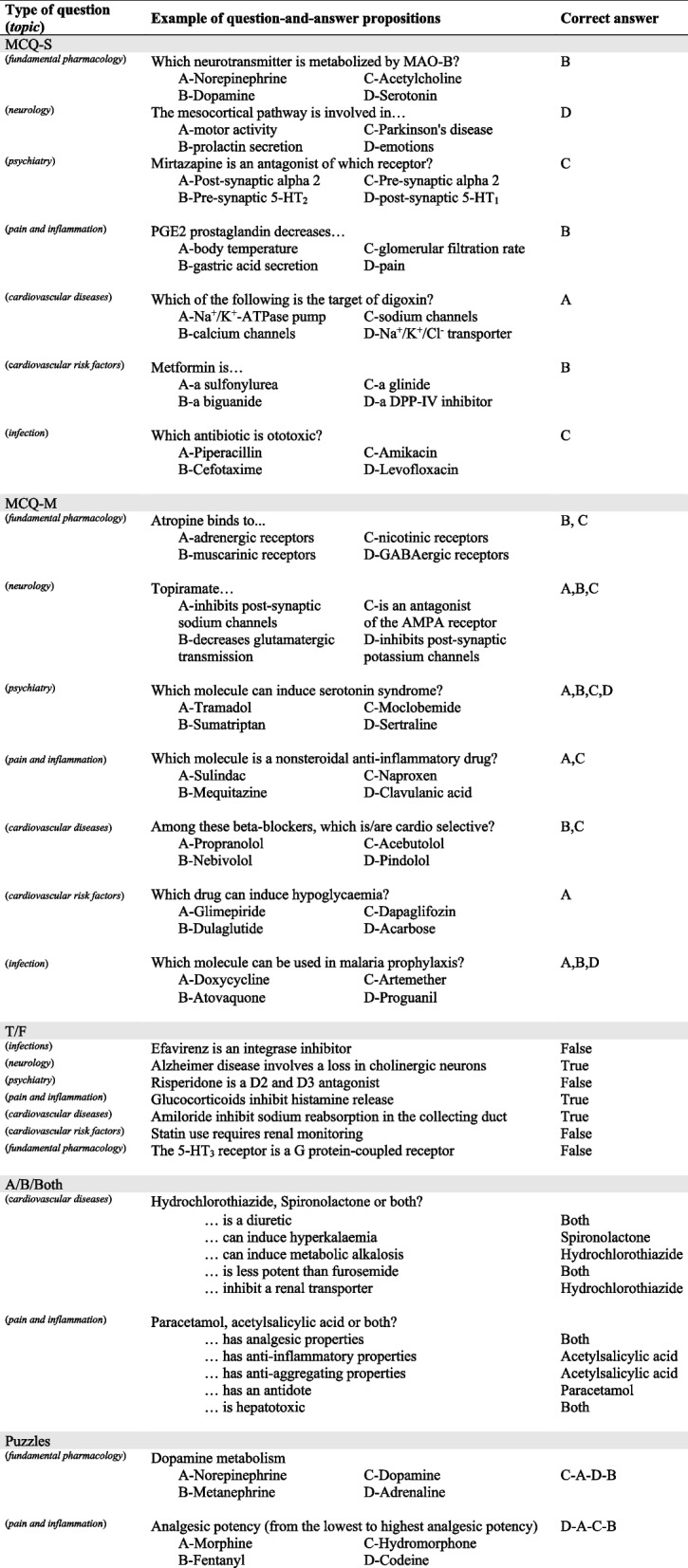
Examples of online questions

*MCQ-S* Multiple choice question with single-select answer, *MCQ-M* Multiple choice question with multi-select answer, *T/F* True or false question

**Table 2:**
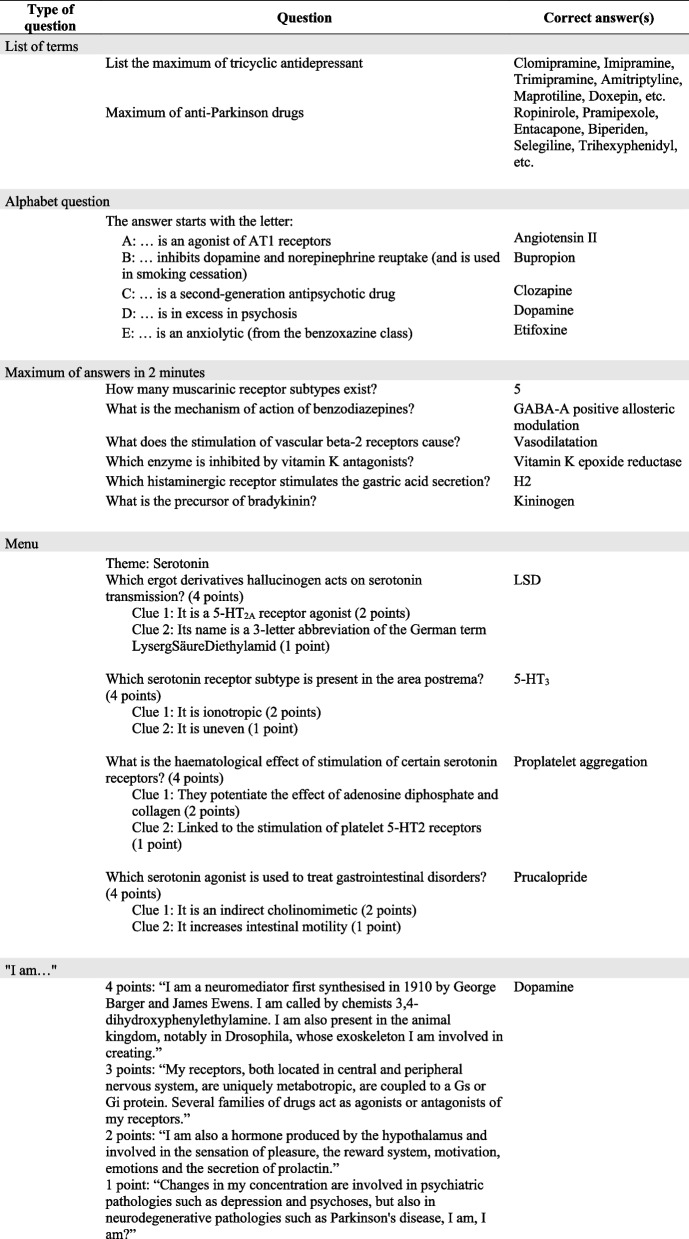
Examples of face-to-face questions

#### Question format

The question formats used in “Pharmacotrophy” differed from those used in pharmacology exams. While exams consist of written questions requiring time for answering and correcting/grading, “Pharmacotrophy” necessitated questions that could be answered swiftly to maintain excitement during matches and allowed to cover a broad field of knowledge. Additionally, these questions needed to be automatically scored and compatible with platforms like Kahoot!®. Thus, questions were inspired by French TV shows and adapted to fit Kahoot!® tool and to provide pedagogical interest. Examples of questions are provided in Tables 1 and 2.

Online questions included:

- Multiple choice questions with single-select answer (MCQ-S): only one of the four alternative answers was correct; players had 30 seconds to respond.
- Multiple choice question with multi-select answers (MCQ-M): one to four of the alternative answers were correct; players had 30 seconds to respond.
- True/False (T/F): players had to answer whether the statement was true or false within 5 seconds.
- A/B/both: two molecules or classes “A” and “B” were given at the beginning of a set of 10 statements; for each statement, participants had to indicate whether it applied to A, B or both within 5 seconds.
- Puzzle: players had to arrange answers in the correct order within 30 seconds.

For single-select questions (MCQ-S, T/F, A/B/Both and puzzles), players could earn up to 1000 points for a correct answer. Multi-select questions (MCQ-M) offered up to 500 points for each correct answer selected. If at least one wrong answer was selected, the player received no point. The number of points earned for a correct answer in any question type decreased based on the speed of the response, reaching half of the maximum points when the response was made at the time limit.

Face-to-face questions included:

- List of terms: players had 1 minute to individually write on a whiteboard as many drugs of a given class or drug classes used to treat a given disease. Each team scored one point for each correct term named.
- Alphabet question: an open question format where the answer had to begin with a given letter. The fastest team to answer correctly earned one point. A wrong answer resulted in the point going to the opposite team.
- Maximum of answers in 2 minutes: each team had to answer, one after the other, as many open or true/false questions in 2 minutes. Teams could pass to the next question without giving an answer and one point was awarded for each correct answer.
- “I am …” : one teacher read a first-person description of a molecule or a drug class. The first team to correctly identify the molecule or drug class scored. The number of points decreased (from four to one) as the description progressed.
- Menu: the team leading at the end of the previous round had to choose within three topics one for themselves and one for the opposite team. Open questions were asked within the topic and granted with four points. Only one answer could be given, a wrong answer resulted in no point. Teams could ask for clues, but this reduced the number of points earned for the correct answer (two points after one clue and one point after two clues).

Regardless of the question type, both online and face-to-face matches included some fun questions to break the seriousness and allow players to relax.

Group stage matches consisted of MCQ-S and MCQ-M. Knockout stage questions included a mix of MCQ-S, MCQ-M, T/F, A/B/both and puzzle questions (Additional file 1). In the 2022 semi-finals, two lists of terms and 26 alphabet questions. The final consisted of a series of maximum of answers per team, a series of 6 “I am...” questions and a menu per team with 6 questions with clues (Additional file 1; Fig. 1).

### Rate of correct answer

The percentage of participants who correctly answered each question was collected after excluding funny questions. These rates were pooled to calculate the rate of correct answers for each type of question.

### Feedback

Participants’ feedback regarding satisfaction, general organisation and opinion on the different types of questions were obtained through an anonymous online form (Additional file 1). As we wanted the most exhaustive feedback from participants, we analysed 4th and 5th year’s feedback together. Teachers’ feedback was also collected through an anonymous online form in 2022.

### Exam mark comparison

To assess the baseline level of pharmacology knowledge, the results of the fundamental pharmacology exam conducted in the first semester of the 3rd year were analysed. This exam included fundamental pharmacology and physiology questions. As the data analysis was conducted retrospectively, the exam sheets were no longer available and only the total exam mark (/20) was available without the ability to differentiate the marks for pharmacology questions (/12) from those for physiology questions (/8). As pharmacology questions had a higher total number of points, we considered the total mark to be representative of the pharmacology level.

Regarding the 4th year, the following data were analysed:

- Final grade average: the average of the final marks of the first and second semesters of all the 4th year examinations. It includes marks obtained in knowledge tests and practical works common to all students (from the core curriculum). It also includes the pre-professional orientation marks (from elective courses), which can be assessed by knowledge tests or the mastery of professional applications, depending on the option/ course chosen.

- Mark in PNEDI courses: the PNEDI (“*Pathologies du SNC, Neuropathies, Endocrinopathies, Douleur et Inflammation”*) course examination is the only knowledge test common to all 4th year students in the second semester. It takes place at the end of the year, after the revision period and a few days after the tournament. It encompasses three teaching units, each with an independent exam: “CNS disorders and neuropathies”, “endocrinopathies” and “pain and inflammation”. The questions cover pharmacology but also physiology, therapeutic chemistry and clinical pharmacy. The mark in PNEDI courses is the mean of the marks obtained in these three examinations, out of 20.

- Mark in pharmacology questions: this represents the sum of the five questions interrogating pharmacology knowledge in the “pain and inflammation” and “CNS disorders and neuropathies” exams of the PNEDI courses, marked out of 18 in total. Three pharmacology teachers wrote the questions in this exam and only one of them (VCB) was involved in the organisation of “Pharmacotrophy”.

- Mark in non-pharmacology questions: this represents the sum of nine questions asked by non-pharmacology teachers in the “pain and inflammation” and “CNS disorders and neuropathies” sections of PNEDI courses, scored out of 22.

For each year and each data listed above, the mean of “Pharmacotrophy” participants and the mean of non-participants in the same class were calculated. Due to variability in question difficulty, and differences in course and exam conditions, especially related to lockdown measures, results from 2021 and 2022 were not pooled for raw mark analysis. Instead, for each student, the ratio of the student’s individual mark to the mean mark of the entire class was calculated. This ratio indicated the proportion of the student’s mark compared to the mean mark of the whole class. The mean of the ratios for “Pharmacotrophy” participants and the non-participants in the same class was then calculated.

The number of students who failed to pass the first exam session and had to retake it in the second session was also calculated. A final grade average of less than 10/20 or a mark < 8/20 in one of the final exams required the student to attend the second session for all exams in which they had a mark < 10/20 before entering the 5th year.

### Statistical analysis

Data are expressed as mean ± standard error of the mean (SEM). The Shapiro-Wilk test was used to evaluate dataset normality. Difficulty and interest scores for students and correct answer rates were compared using one-way ANOVA followed by Tukey’s multiple comparisons. Standard deviations were compared using Bartlett’s test. Correlation between interest and difficulty were made using the Pearson correlation test. Comparisons of marks/ratios were performed using Student t test(^a^) or Mann Whitney test(^b^) when samples did not follow a normal distribution. Success in the first session was analysed using a Chi-square test. Comparison of marks according to progression in “Pharmacotrophy” was conducted using one way ANOVA or a Kruskal Wallis test followed by post-hoc tests, Student t test(^a^) or Mann Whitney test(^b^) respectively. Values of probability lower than 5% (*p* < 0.05) were considered significant.

### Ethics and consent

According to the French legislation, submission to an ethic committee was not mandatory for our study. The participation in the event and in the online questionnaire was voluntary. Students were free to leave the event at any time. When filling the registration form and the feedback questionnaire, students gave their informed consent for their data to be used anonymously for communication and research purposes only.

## Results

### Participants

In 2021, a total of 27 students took part in the “Pharmacotrophy”, 20 were in 4th year (74%) and 7 in 5th year (26%). Of the 9 teams, 6 were composed exclusively from 4th year students and 1 was mixed. In 2022, 36 students participated (+ 33% compared to 2021), 22 were in 4th year (61%), 13 in 5th year (36%) and 1 in 6th year (3%). Of the 12 teams, 5 were composed exclusively from 4th year students, 5 were mixed and 2 had only 5th year. In 2022, among all participants, 26 (72%) stated on the registration form that they joined for the challenge, 24 (67%) to have fun and 25 (69%) for educational interest. Among these 25, 20 said they joined to learn through the questions asked, and 16 to incite them to revise before matches.

### Participants’ feedback

#### Respondents

In 2021, a feedback form was completed by 21 participants (78%). In 2022, 28 participants (78%) provided feedback.

#### Satisfaction

In 2021, 100% of the respondents were satisfied with their participation in “Pharmacotrophy” with 95% (20/21) being very satisfied (maximum positive evaluation). All participants found the event quite or very satisfactory to revise, to have fun or to have social interaction in the lockdown context. In contrast, 14% of the respondents (3/21) found “Pharmacotrophy” unsatisfactory to learn through the questions asked.

In 2022, participants rated their overall satisfaction with “Pharmacotrophy” at an average of 9/10 (min.: 7; max.: 10), with higher scores among participants who made it to the final (group stage: mean = 8.6/10, *n* = 8; quarter- and semi-finals: mean = 9/10, *n* = 14; final: mean = 9.5, *n* = 6). All students found “Pharmacotrophy” satisfactory for entertainment (85% very satisfactory) and educational purposes (89% very satisfactory): 93% to learn through questions, 89% to revise before matches and 89% to revise after matches.

Open comments widely highlighted the educational advantage of learning while having fun (for example: “*A great way to evaluate your knowledge and appreciate pharmacology from another perspective than the courses, the best motivation to work and to finally retain that damn course on fibrates*”). Some students pointed out that “Pharmacotrophy” provided a proximity to teachers and a desire to learn and succeed in exams (for example: “*Often in a large Faculty like Paris, you don’t have the proximity with the professors and you feel a bit alone. This kind of initiative makes it possible to create proximity with the students. The contact with the teachers is so motivating, we want to learn and to see people so involved motivates us to pass our exams*”). Many students expressed a desire for more teachers to offer such events. The informal atmosphere of the event was also appreciated. The fun aspects, such as teachers’ costumes, music, and funny questions, were among the aspects that most participants (75%) wanted to keep for the next year.

At the end of the tournament, the majority of respondents in both 2021 (76%) and 2022 (82%) expressed their willingness to participate again the following year. When asked how much they would recommend “Pharmacotrophy” to other students next year, 86% (18/21) of participants gave a score of 5/5 and 14% (3/21) a score of 4/5 in 2021. In 2022, 79% (*n* = 22) gave a score of 5/5, 14% (*n* = 4) a score of 4/5 and 7% (*n* = 2) a score of 3/5.

#### Overall organisation

**Fig. 2:**
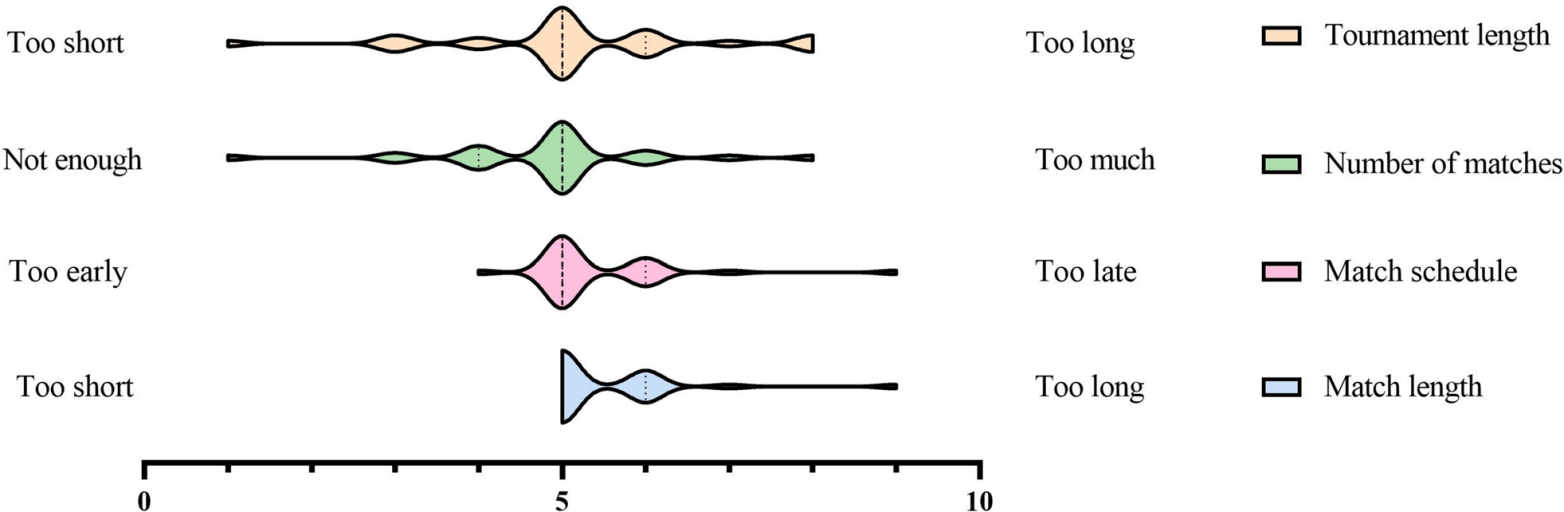
Students’ evaluation of the overall organisation of 2022 “Pharmacotrophy” (*n* = 28)

The majority of students felt that the tournament’s length was adequate and that they had played a sufficient number of matches (Fig. 2). However, those who felt that they had not played enough matches were mostly eliminated in the group stages, while those who felt they had played too many matches made it to the knockout stage. The matches schedule seemed appropriate, and the period of the year seemed ideal as all courses were recently finished and the tournament hold not too late to interfere with revision.

#### Matches

In 2022, participants were asked for their opinion on the format of the matches. Regarding online matches, they appreciated having the wide range of topics that covered the whole pharmacology curriculum. Some students regretted the lack explanations for the questions during or after the matches, which could have helped them integrate the knowledge better. The increase in question variety and difficulty for the knockout stage was appreciated. The play-off match, with a larger number of participants and more questions, was considered the most fun and challenging.

Out of the 12 respondents who played a face-to-face match, none of them preferred online matches. Five preferred face-to-face and seven found both kind of matches equally good. They really enjoyed the possibility of discussing with their team and answering cooperatively. The direct competition with the opposing team through speed-based questions added pressure and stimulation to the match and was appreciated by students.

#### Questions

In 2022, among the online questions (Table 3), the interest did not significantly vary depending on the type of question (p_ANOVA_ = 0.08). On the contrary, the difficulty perceived differed depending on the question type (p_ANOVA_ < 0.0001). Puzzle questions, which were both reported challenging and confusing, were rated significantly more difficult than the MCQ-S (*p* < 0.0001), MCQ-M (*p* = 0.0181), T/F (p < 0.0001) and A/B/Both (*p* = 0.0003). MCQ-M were also perceived more difficult than MCQ-S (*p* = 0.0249) and T/F (p < 0.0001).

**Table 3:**
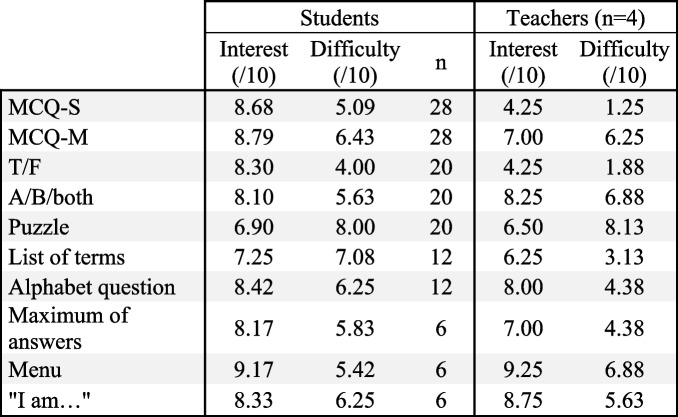
Students’ and teachers’ evaluation of interest (0 indicating no interest and 10 indicating the highest level of interest) and perceived difficulty (0 indicated no difficulty and 10 indicated the highest level of difficulty) for different type of questions, in 2022

*MCQ-S* Multiple choice question with single-select answer, *MCQ-M* Multiple choice question with multi-select answer, *T/F* True or false question

Regarding face-to-face questions, fewer students were able to participate which impedes statistical comparison and requires caution in interpretation. The list of terms was considered as the most difficult and the less interesting. Conversely, the menu was considered the easiest and the most interesting (Table 3).

**Fig. 3:**
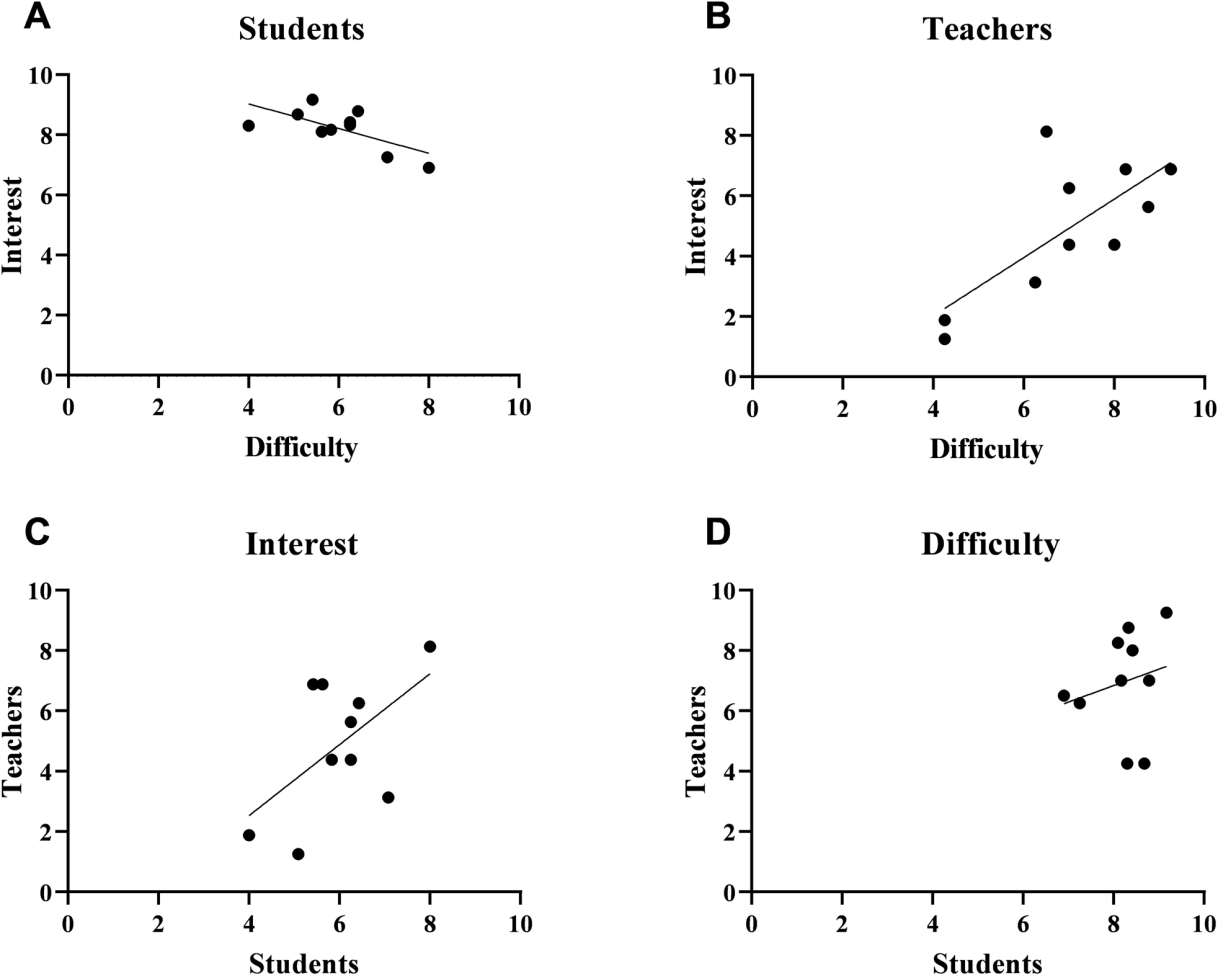
Pedagogical interest and perceived difficulty for students and teachers in different question types (*n* = 10) in 2022 Each plot corresponds to the mean score for a type of question. Correlations were made using Pearson’s correlation test. *A: r* = − 0.2762, *p* = 0.0375; *B: r* = 0.7296, *p* = 0.0166; *C: r* = 0.5659, *p* = 0.0881; *D: r* = 0.2158, *p* = 0.5494

After pooling the individual answer of all participants for all type of question (*n* = 158), interest was negatively correlated to difficulty (r = − 0.2762; *p* = 0.0004; data not shown). Considering the mean participant’s score for each type of question, interest is still negatively correlated to difficulty (r = − 0.6609; *p* = 0.0375; Fig. 3A).

### Teachers’ feedback

The teachers who organised “Pharmacotrophy” expressed a high level of appreciation for their involvement, giving an average satisfaction score of 9/10 (min: 8; max: 10). They observed that “Pharmacotrophy” fostered a closer connection between students and teachers compared to traditional lectures. They found this novel approach to pharmacology, devoid of marks or judgement and set in a relaxed environment, very positive for the students. Interestingly, teachers also found that organising “Pharmacotrophy” and elaborating diverse question types had a formative effect on their own teaching methods, prompting them to approach their courses differently.

Teachers perceived the tournament duration as slightly lengthy (mean 7.5/10, with 1 as “too short” and 10 as “too long”). The organisation of “Pharmacotrophy” required a considerable amount of effort (mean: 9.25/10; min.: 8; max.: 10). They estimated investing approximately 2 hours per day in video conferencing (for match animation, debriefing after matches and preparation for the next day’s matches), and an additional 2 to 4 hours per day to prepare questions and matches per teacher. Over the 9 days of competition, this amounted to a workload of 36 and 54 hours of work per teacher, i.e. between 144 and 216 hours of total work.

Teachers found online matches to be easier to organise and manage. However, they expressed that face-to-face matches were more intense, playful and dynamic as they allowed discussion and interaction among team members.

From an educational perspective, teachers found MCQ-S and T/F to be the easiest and least interesting for students. On the other hand, menu questions were considered the most interesting for students and puzzles were considered the most difficult. While face-to-face questions were considered more enjoyable to create, they were also more difficult to elaborate compared to online questions (Additional Fig. 1).

After pooling individual answers of all teachers for all type of question (*n* = 40), it appeared that interest was positively correlated to difficulty (r = 0.5239; *p* = 0.0005; data not shown). Considering the mean teacher’s score for each type of question, interest was still positively correlated to difficulty (r = 0.7296; *p* = 0.0166; Fig. 3B).

### Comparison between participant’s and teacher’s feedback

Perceived pedagogical interest (r = 0.5659; *p* = 0.0881) and difficulty (r = 0.2158; *p* = 0.5494) was not correlated between teachers and students (Fig. 3C and D).

### Rate of correct answer

The mean proportion of students providing a correct answer was calculated for each type of online question. Mean rates for each type of question did not differ between 2021 and 2022 (data not shown). We thus pooled data of both years to get 404 MCQ-S, 173 MCQ-M, 120 A/B/both, 104 T/F and 30 puzzles. Rates are shown in Table 4.

**Table 4:**
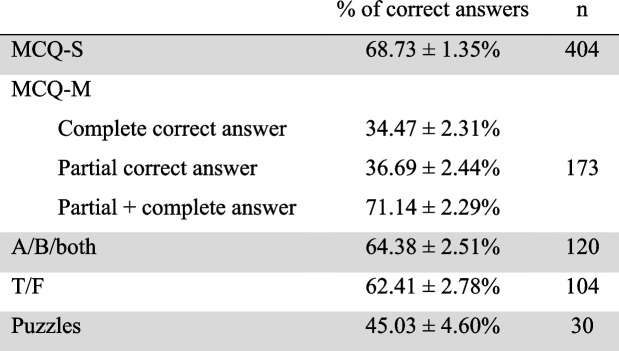
Rate of correct answers of the different type of question over 2021 and 2022

*MCQ-S* Multiple choice question with single-select answer, *MCQ-M* Multiple choice question with multi-select answer, *T/F* True or false question

The rate of correct answer differed depending on the type of question (p_ANOVA_ < 0.01). Especially, the rate of complete correct answer with MCQ-M was lower than with MCQ-S (*p* < 0.01), A/B/both (p < 0.01) and T/F (*p* = 0.01). Rates were also lower with puzzles than MCQ-S (p < 0.01), A/B/both (p < 0.01) and T/F (*p* = 0.02). Other rates did not significantly differ.

When considering only complete correct answers for the MCQ-M, the rate of correct answers per type of question was not correlated to student’s or teacher’s perceived interest or difficulty (data not shown; Table 5). Interestingly, when considering partial answer also as correct answers for MCQ-M, the rate of correct answers became correlated to student’s perceived interest but was still not correlated to the other parameters (data not shown; Table 5).

**Table 5:**
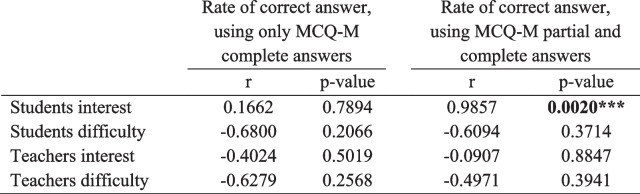
Correlation coefficient between the rate of correct answers and the other parameters when considering only complete correct answers or partial and correct answers for MCQ-M

Tests were made using Pearson correlation test

***p < 0.01

### Exam mark comparison

#### Students

Among the 20 participants in 4th year in 2021, 1 did not pass the 4th year exams and was excluded from the mark analysis. In 2022, among the 22 participants in 4th year, 2 doubled their 4th year and had already validated some of their exams. They were also excluded from the mark analysis. The number of other students in the 4th year class varied between 315 and 320 in 2021 depending on the exam, as some repeaters did not retake all the exams. In 2022, between 279 and 281 4th year students did not participate in “Pharmacotrophy”.

#### Comparison of marks in 3rd year

In 2021, students who participated in “Pharmacotrophy” had a mean mark of 10.67 ± 0.62 (*n* = 19) in the exam containing fundamental pharmacology questions in the 3rd year, which was similar to the mean mark of 10.23 ± 0.20 (*n* = 306) obtained by the rest of the class (*p* = 0.6416^b^). Likewise, in 2022, 3rd-year participants achieved marks of 11.61 ± 0.43 (*n* = 20), which were similar to the mean mark of 10.73 ± 0.12 (*n* = 339) obtained by the rest of the class (*p* = 0.1033 ^b^).

#### Comparison of marks and general level in the 4th year

The general level of students, as assessed by the final grade average, was similar among participants and non-participants in “Pharmacotrophy” for both 2021 and 2022, as well as when considered the combined ratio from both years (Table 6). The proportion of students who failed the first session was also similar across all groups (Table 7).

**Table 6:**
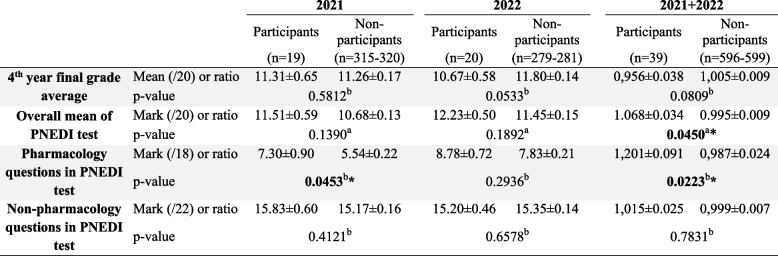
Mark comparison between participants and non-participants at the 4^th^ year exams, after Pharmacotrophy

Mean mark of 4^th^ year students who participated in Pharmacotrophy compared to that of the rest of the 4^th^ year class (non-participants) in 2021 and 2022 and ratio of the individual mark to the mean class mark for 2021 + 2022. Data are expressed as mean ± SEM

*p < 0.05

^a^Student t test

^b^Mann Whitney test

**Table 7:**
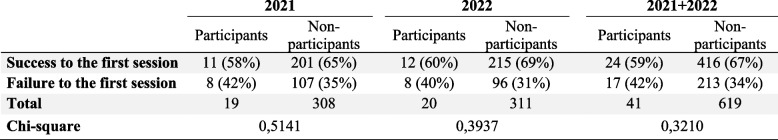
Proportion of success in the first session according to participation in Pharmacotrophy

Regarding PNEDI mark, which is the only knowledge test for the 4th year’s second semester, students who participated in “Pharmacotrophy” obtained better results than non-participants when considering both years together. This difference was not significant when comparing each year individually (Table 6).

Regarding the level of pharmacology questions in the PNEDI test, “Pharmacotrophy” participants had better marks than non-participants in 2021 but not in 2022. Over 2021 and 2022, the ratio of the individual mark to the class mean was significantly higher for participants than for the rest of the class (Table 6). Thus, on average and over both years, “Pharmacotrophy” participants had 20.1% higher marks on pharmacology questions than non-participants. These results were consistent when comparing the sum of pharmacology question marks (data not shown), suggesting no bias related to the teacher asking the questions.

Regarding non-pharmacology questions, marks were similar between participants and the rest of the class in 2021, in 2022 and when considering the ratio over both years (Table 6).

#### Comparison according to progression

After splitting the participants depending on their progression in “Pharmacotrophy”, samples were too small for a relevant statistical analysis of each year individually (only two 4th year students made it to the final in 2021, and only two were eliminated during the group stage in 2022). Ratios of the individual mark to the mean class mark were thus combined.

**Fig. 4:**
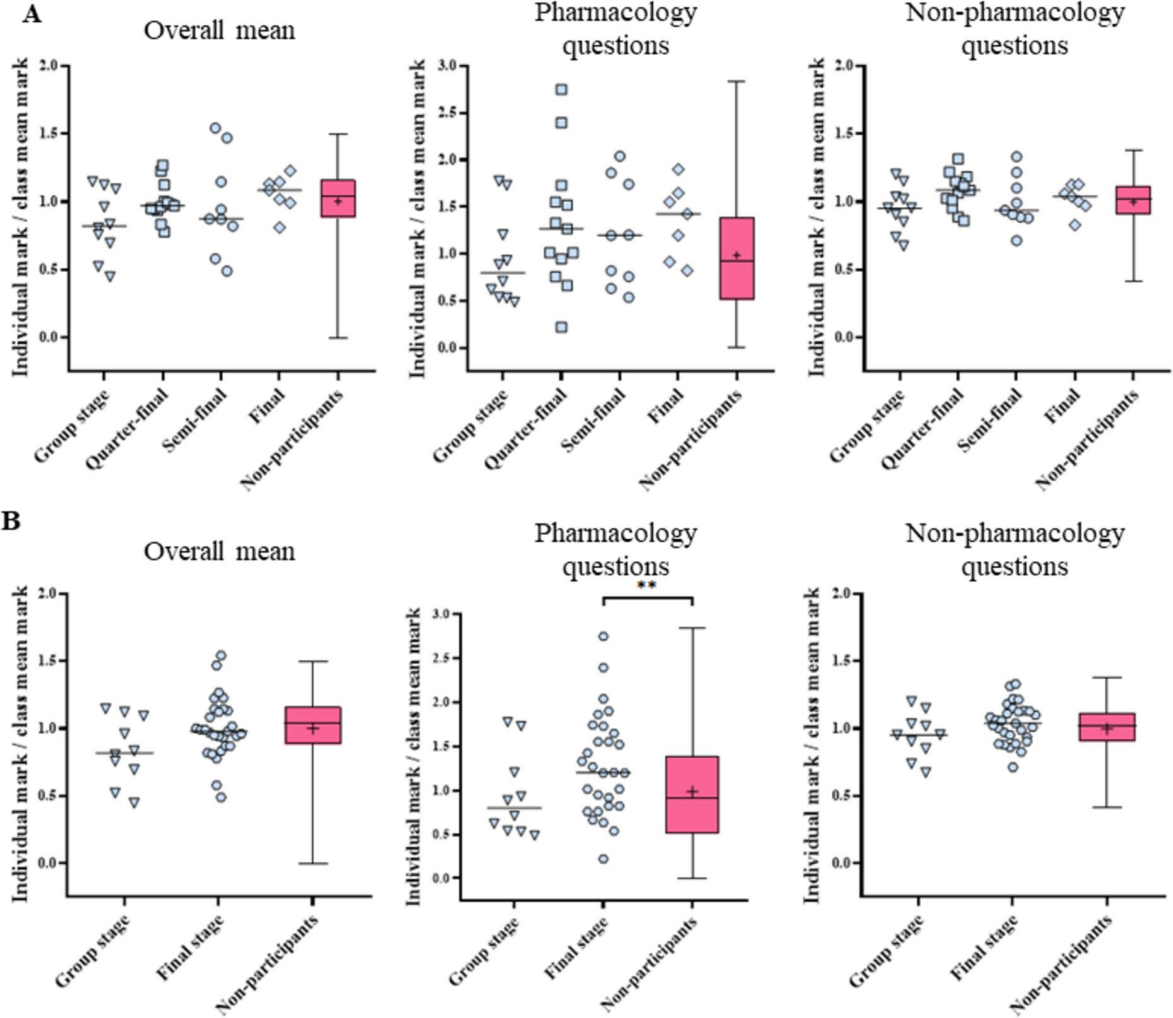
Ratios of the individual mark to the class mean in the PNEDI exam, depending on the progression in “Pharmacotrophy” Final-stage reached detailed (A) or pooled (B). “Overall mean” correspond to the overall PNEDI mark, which combines the mark for the pharmacology and non-pharmacology questions. Means are indicated by a horizontal bar or “+”. Comparisons were performed using Kruskal-Wallis test. n_Group stage_= 10, n_Quarter-final_= 13, n_Semi-final_= 9, n_Final_= 7, n_Final stage_= 29, n_Non-participants_= 596-599.**p < 0.01

The final grade average and the mark on pharmacology or non-pharmacology questions in the PNEDI test did not significantly depend on the progression in “Pharmacotrophy” (*p* = 0.2089, *p* = 0.0908 and *p* = 0.4109, respectively) (Fig. 4A).

To increase the statistical power, all students who reached the final stage were grouped together. The final grade average tended to increase with progression, but not significantly (*p* = 0.0881) (Fig. 4B). Regarding pharmacology questions, marks varied with progression (*p* = 0.0221). Students reaching the final stage obtained better marks than the rest of the class (1.290 ± 0;107 vs 0.986 ± 0.024; *p* = 0.0057^b^). No significant difference was observed between students eliminated in the group stage and the rest of the class or between students eliminated in the group stage and those who reached the final stage (Fig. 4B). On the other hand, regarding non-pharmacology questions, marks did not vary according to the progression (*p* = 0.3553) (Fig. 4B).

## Discussion

Besides describing the organisation of “Pharmacotrophy”, the objectives of this study were twofold: (1) to assess the feedback from both students and teachers, and (2) to evaluate the participants’ performance in exams compared to non-participants. The results indicated that students’ feedback was overwhelmingly positive, and there were indications of a positive effect of participating in “Pharmacotrophy” on success in pharmacology exams.

### Effects on behavioural changes

Both qualitative and quantitative analyses of students’ feedback indicated the successful achievement of our study first objective: “Pharmacotrophy” motivated students to engage with and transform their perception of pharmacology through enjoyable revision sessions. All participants reported satisfaction and educational interest. They reported how “Pharmacotrophy” positively influenced their approach to pharmacology, making the revision process enjoyable and providing pedagogical benefits. These findings align previous studies utilising GBL [6, 36], especially those using Kahoot!® or online quiz [57–59] or similar friendly competition [49]. We can assume that “Pharmacotrophy” fostered pharmacology revisions with both intrinsic motivation stemming from enjoyable and fascinating learning experiences during matches, and extrinsic motivation driven by the desire to win the tournament. This approach contrasts with solely relying on extrinsic motivation linked to exam success [60–62]. Beyond facilitating course revisions, boosting intrinsic motivation has been shown to enhance student performance [63, 64].

A reward system, including points for correct answers, progression in the tournament and a final prize-giving ceremony shared on social networks, further enhanced motivation and engagement. This system, linked to the theory of reinforcement [65], yielded positive effects on GBL outcomes [32], with observed long-term benefits in medical education [66–69].

The incorporation of fun elements, such as TV show based questions, music, costumes, offbeat questions, team progression and collaborative learning, contributed to a positive learning environment [57, 58], promoting learning-related functional changes in the brain [65, 70] beneficial to learning process [38–40]. Additionally, the event’s informal atmosphere fostered a closer connection between students and teachers. This was reported as beneficial from students, but also from teachers, as it provided insights into how students perceived pharmacology courses, as reported by others [71].

Many studies have shown that repetitive exposure to questions after incorrect answers effectively enhances knowledge acquisition [72–74]. However, our approach diverged from this by prioritizing diverse question types to stimulate reflection, avoiding rote memorization. This aligns with the “mediator effectiveness” hypothesis [75], which suggests that varied active retrieval of information fosters a multifaceted understanding, consistent with learning principles [65, 75].

Face-to-face matches facilitated skill-sharing and promoted social learning [76–78], a major component of GBL [39, 57] that shown educational benefits [52, 79]. While online questions lacked collaborative reflection, combining scores and distributing responsibilities balanced competition’s negative aspects [65, 80], fostering learning communities and enhancing interpersonal skills [65].

Immediate feedback is crucial in deliberate practice theory [18]. While most GBL provides answer explanation immediately [38, 81], our competition format focused on indicating correct answer without disrupting the game’s flow, still proving beneficial according to similar competitive quiz contexts [27, 49, 82].

“Pharmacotrophy” integrates elements from CBL into its tournament modelled on a sports competition. CBL adds motivation, engagement, and fun, thus promoting social learning [49, 82–86] and enhancing GBL effects [6] and knowledge acquisition [80, 83]. Ranking and comparison among peers is a powerful motivational factors [82, 83], allowing them to assess their performance to each other [87] and identify areas for improvement [88]. Mixing participants from different academic years in the tournament might have promoted inter-year competition (between teams of different academic years) or collaboration (when participants were mixed in the same team), and probably enhanced the tournament’s social dimension. However, caution is needed with CBL to avoid a stressful atmosphere which could decrease fun, motivation and engagement. Anonymity is thus an essential element to mitigate adverse effects [83, 89].

### Effect on knowledge acquisition

We showed that participants in “Pharmacotrophy” had better marks in pharmacology compared to non-participants. However, to state with confidence that students are better in pharmacology specifically because of their participation in “Pharmacotrophy”, we have to exclude two major possible biases. A first selection bias in our study could have been that participants were simply the best of their class, which would have explained their higher marks. We therefore compared the participants to the rest of the class on their final grade average for the whole year, which reflects the student’s level, and on their rate of success in the first session. Participants had similar final grade average and even a lower pass rate in the first session, although not significant. Thus, they seemed to have the same general level than the rest of the class and they were specifically better in pharmacology. A second selection bias could have been that participants were better in pharmacology at baseline, before “Pharmacotrophy”. We therefore compared the students’ marks to the previous year’s tests which included pharmacology questions. Our investigations indicated that the year before “Pharmacotrophy”, participants were not better in pharmacology than the rest of the class. Although the state of knowledge of the students may have changed in 1 year, these data argue for an equivalent level of knowledge among students before “Pharmacotrophy”.

Finally, students who went furthest in “Pharmacotrophy” were better in pharmacology than the other participants. Our results do not allow to differentiate whether they had better results in pharmacology because they had more matches to prepare and play or whether they were better in pharmacology at baseline, which helped them to progress further in the competition. Conversely, as the students eliminated early in the competition seemed to have a lower level than the others, “Pharmacotrophy” would be helpful to identify before the exams the students who have the most difficulty with pharmacology. Thus, we could offer them support sessions in preparation for the exams. However, this last assumption must consider two elements. First, the main format of “Pharmacotrophy” question was MCQs which, although efficient for covering a broad range of topics, may not fully reflect students’ abilities in reflection and deep understanding [90, 91]. That is the reason why written questions are preferred for the exams. This mismatch in question format could lead to discrepancies between students’ performance in “Pharmacotrophy” and their performance in exams. On the other hand, the different format of question guarantees that performance in exams is truly a result of knowledge acquisition rather than mere familiarity with exam questions. Second, the heterogeneity of level in students of the same team. Regarding pharmacology question marks, some students were well above the mean of the students eliminated at the same stage (e.g. two students eliminated in group stage and two in third/quarter) (Fig. 4). This may be due to the fact that they were teamed with students with a lower level in pharmacology. As the team score is calculated as the sum of the scores of each participant, their progression in “Pharmacotrophy” does not necessarily reflect their level in pharmacology.

### Questions analysis

The relationship between difficulty and interest in the questions differed between teachers and students. For students, interest decreased as difficulty increased, while the opposite was true for teachers. Consequently, there was no correlation between teachers’ and students’ assessments of question difficulty. However, there seemed to be some alignment in their evaluation of question interest, though not statistically significant. It is worth noting, that while the rate of correct answers did not seem to influence perceived difficulty for either students or teachers, it might have increased student interest. This become particularly relevant when considering the MCQ-M. Indeed, despite having, by far, the lower rate of complete correct answers, students perceived MCQ-M as easier than others like puzzles, and the most interesting of all. However, when considering the partially correct answers, MCQ-M had the highest correct answer rate among all question types. This leads to two hypotheses. First, students might have considered the rate of partial correctness in their assessment of question difficulty. Secondly, students’ interest may have been more closely linked to scoring and rewards than solely providing the complete correct answer. With Kahoot!®'s current scoring system on partial correct answers, the MCQ-M allow for selecting top performers without excessive discrimination, as all participants generally scored points and received positive feedback, fostering the reward process.

### Limitations

The main limitation of our study is that we cannot state with complete certainty that “Pharmacotrophy” participants were not better than the rest of the class before the event. Although the 3rd year results seem to indicate a similar baseline level, the *p*-value for the comparison of the 2022 participants is quite low for such a small sample and this does not necessarily imply an equivalent level 1 year later. In addition, the only way to accurately determine that the progression is due to the participation in “Pharmacotrophy” would be with a randomised controlled study with students willing to participate in the tournament randomly assigned in the participant group or in the control group. However, such an approach would raise ethical concerns, as it would deprive volunteer students from the opportunity to participate in the event potentially jeopardizing their chances of succeeding in exams.

Our study suffers from small samples and high variability to proceed to more specific analyses of certain results. Especially, we cannot assess precisely whether the fact of having participated in the semi-finals and the final in 2022 had a greater effect on knowledge acquisition. Indeed, these matches were the only ones that took place in person and that allowed collective reflection. This type of reflection has been shown to promote learning [50, 53]. If we had confirmed this, we could have revised the organisation to seek to integrate more face-to-face matches.

### Perspectives

Feedback from students highlighted areas for improvement in the organisation of “Pharmacotrophy”. Some students felt the tournament too long, particularly those reaching the final rounds, while early eliminated did not find it too short. It is noteworthy that teams eliminated early may benefit less from the reward effect. Additionally, by the second week, less than half of the initially engaged students remain, likely including the most skilled in pharmacology, for whom the tournament may have fewer pedagogical benefits. A revised could involve increasing group stage matches and reducing knockout stage, supported by research favouring short, high-energy competition for constructive CBL environments [92].

Involving students in question development could promote experiential learning [65] and save teachers’ time while benefiting students pedagogically [40, 81, 93]. Collaboration, valued by students [39, 57], should be encouraged through collaborative questions. Face-to-face matches are dynamic but lack anonymity. Presenting Kahoot!® questions in an amphitheatre format, with teams collectively answering, could address this [57].

“Pharmacotrophy” lasted 2 weeks, yet studies advocate for spaced-education and multiple weekly session for weeks or months [72–74, 83, 86, 94]. Offering Kahoot!® questions post-event, as requested by students and done in similar competitions [57] could be beneficial.

## Conclusion

“Pharmacotrophy” provided students with an enjoyable way to review pharmacology coursework, fostered a stronger bond between pharmacology teachers and students, and reignited the interest in pharmacology for some. The event’s informal and entertaining atmosphere, with costumes, music, and amusing questions, played a significant role in its success. Additionally, the inclusion of gifts and the presence of supporters further contributed to students’ satisfaction. The tournament proved to be a valuable tool for students during the revision period, allowing them to review the entire curriculum in an engaging and playful manner. Our study also revealed that participation in this educational tournament positively impacted knowledge acquisition, leading to better performance in pharmacology exams. These promising findings serve as a strong motivation to continue and expand this pedagogical initiative, especially for students with the greatest learning difficulties in pharmacology. We are considering extending “Pharmacotrophy” to include more disciplines and potentially involving other universities, with the aim of transforming it into a national competition.

### Supplementary Information

**Supplementary Material 1.**

## Data Availability

The datasets used and/or analysed during the current study available from the corresponding author on reasonable request.
