# Supplementary material for: “Pharmacotrophy”: a playful tournament for game- and team-based learning in pharmacology education - assessing its impact on students’ performance
Source: BMC Med Educ. 2024 Mar 1;24:219. doi: 10.1186/s12909-024-05157-z (PMC10908103; doi:10.1186/s12909-024-05157-z)
Supplement: Supplementary file 1 — Supplementary Material 1. [file 12909_2024_5157_MOESM1_ESM.docx]

**ADDITIONAL METHODS**.

**Additional Table 1.** Organization of matches: frequency of match per stage (group, play-off, quarter-finals, semi-finals, final) and per day; frequency of question type per match; and characteristics of question in terms of response time and collaborative nature.

|  | **2021** | | | | **2022** | | | | |  | |  |  |
| --- | --- | --- | --- | --- | --- | --- | --- | --- | --- | --- | --- | --- | --- |
|  | **Group** | **1/4** | **1/2** | **Final** | **Group** | **Play-off** | **1/4** | **1/2** | **Final** |  | | | |
| **Overall organisation** |  |  |  |  |  |  |  |  |  |  | | | |
| Number of days | 5 | 2 | 1 | 1 | 4.5 | 0.5 | 2 | 0.5 | 0.5 |  | | | |
| Total number of matches | 18 | 6 | 1 | 1 | 18 | 1 | 4 | 2 | 1 |  | | | |
| Number of teams involved | 9 | 2 | 3 | 2 | 12 | 6 | 8 | 4 | 2 |  | | | |
| Teams per match | 2 | 2 | 3 | 2 | 2 | 6 | 2 | 2 | 2 |  | | | |
| Match per team | 4 | 2 | 1 | 1 | 3 | 1 | 1 | 1 | 1 | **Question details** | | | |
| **Number of questions per match** |  |  |  |  |  |  |  |  |  | Response time | Collective discussion | |  |
| *Online* |  |  |  |  |  |  |  |  |  |  |  | |  |
| MCQ-S | 15 |  | 10 |  | 10 | 20 |  |  |  | 30s |  | |  |
| MCQ-M |  |  |  | 24 | 5 | 10 | 15 |  |  | 30s |  | |  |
| T/F |  | 10 | 20 |  |  |  | 10 |  |  | 5s |  | |  |
| A/B/both |  | 10 | 20 |  |  |  | 10 |  |  | 5s |  | |  |
| Puzzle |  |  |  | 10 |  |  | 5 |  |  | 30s |  | |  |
| *Face to face* |  |  |  |  |  |  |  |  |  |  |  | |  |
| List of terms |  |  |  |  |  |  |  | 2 |  | 1min | + | |  |
| Alphabet question |  |  |  |  |  |  |  | 26 |  | SB | + | |  |
| Maximum of answers (set/team) |  |  |  |  |  |  |  |  | 1 | 2min | + | |  |
| "I am…" |  |  |  |  |  |  |  |  | 6 | SB | ++ | |  |
| Menu (questions/team) |  |  |  |  |  |  |  |  | 6 | No limit | +++ | |  |

*1/2: semi-final; 1/4: quarter-final; MCQ-S: multiple choice question with single-select answer; MCQ-M: multiple choice question with multi-select answer;* *min: minute; s: second; SB: speed based (the first team to press the buzzer can give the answer); T/F: true or false question.*

**ADDITIONAL RESULTS**

**Additional Figure 1**. Teachers' point of view on the fun, difficulty, enjoyment and formative aspect of creating different types of questions


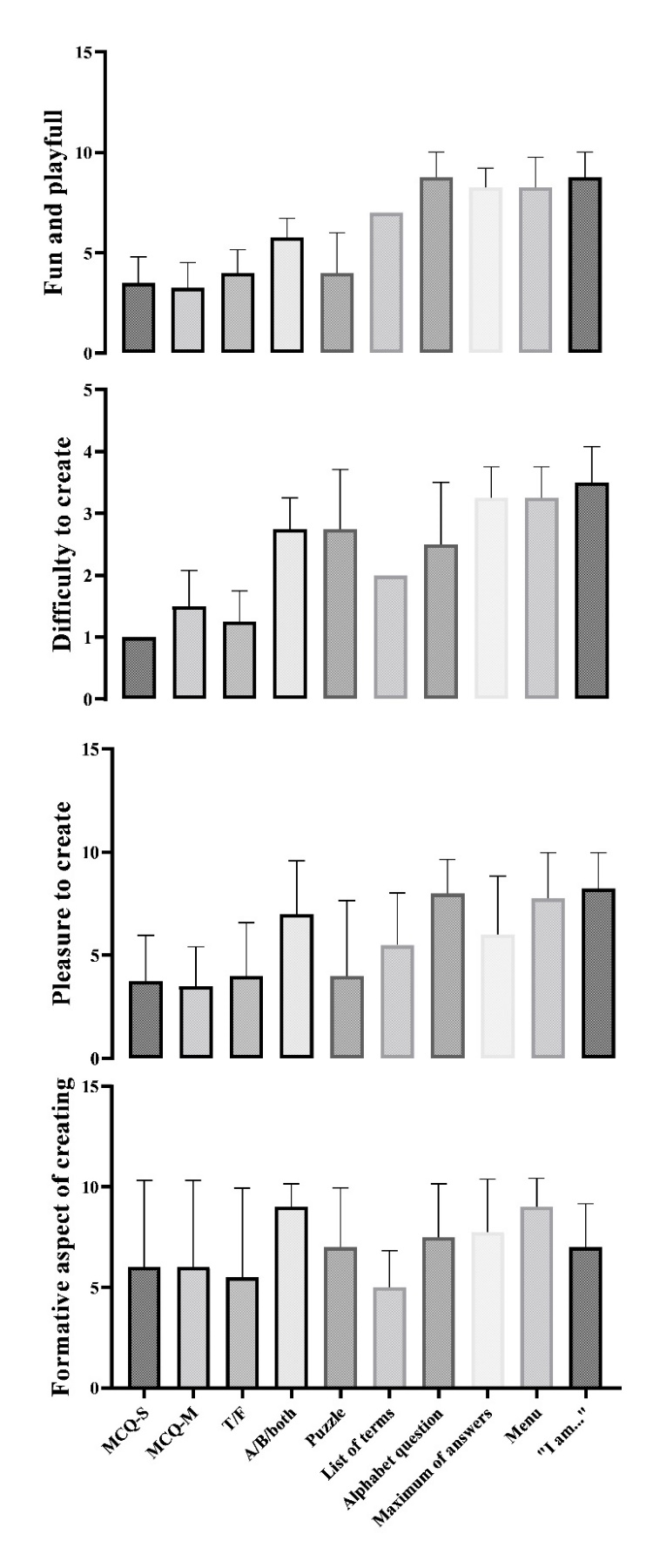


*MCQ-S: multiple choice question with single-select answer; MCQ-M: multiple choice question with multi-select answer;* *T/F: true or false question.*
